# A patient-centered, coordinated care approach delivered by community and pediatric primary care providers to promote responsive parenting: pragmatic randomized clinical trial rationale and protocol

**DOI:** 10.1186/s12887-018-1263-z

**Published:** 2018-09-04

**Authors:** Jennifer S. Savage, Samantha M. R. Kling, Adam Cook, Lindsey Hess, Shawnee Lutcher, Michele Marini, Jacob Mowery, Shannon Hayward, Sandra Hassink, Jennifer Franceschelli Hosterman, Ian M. Paul, Chris Seiler, Lisa Bailey-Davis

**Affiliations:** 1 Department of Nutritional Sciences, Center for Childhood Obesity Research, 129 Noll Laboratory, The Pennsylvania State University, University Park, PA 16802 USA; 2 Geisinger Obesity Institute, Epidemiology and Health Services Research Geisinger, 100 N Academy Ave, Danville, PA USA; 3 Maternal and Family Health Services, 15 Public Square, Suite 600, Wilkes-Barre, PA 18701 USA; 4 Institute for Healthy Childhood Weight, American Academy of Pediatrics, 2602, Pennington Dr., Wilmington, DE 19810 USA; 5 Pediatrics, Public Health Services, Penn State College of Medicine, HS83, 500 University Drive, Hershey, PA 17033 USA

**Keywords:** Early obesity prevention, Responsive parenting, Health information technology, Coordination of care, The special supplemental women, Infants, And children program, Clinical care

## Abstract

**Background:**

Economically disadvantaged families receive care in both clinical and community settings, but this care is rarely coordinated and can result in conflicting educational messaging. WEE Baby Care is a pragmatic randomized clinical trial evaluating a patient-centered responsive parenting (RP) intervention that uses health information technology (HIT) strategies to coordinate care between pediatric primary care providers (PCPs) and the Special Supplemental Nutrition Program for Women, Infant and Children (WIC) community nutritionists to prevent rapid weight gain from birth to 6 months. It is hypothesized that data integration and coordination will improve consistency in RP messaging and parent self-efficacy, promoting shared decision making and infant self-regulation, to reduce infant rapid weight gain from birth to 6 months.

**Methods/design:**

Two hundred and ninety mothers and their full-term newborns will be recruited and randomized to the “RP intervention” or “standard care control” groups. The RP intervention includes: 1) parenting and nutrition education developed using the American Academy of Pediatrics Healthy Active Living for Families curriculum in conjunction with portions of a previously tested RP curriculum delivered by trained pediatric PCPs and WIC nutritionists during regularly scheduled appointments; 2) parent-reported data using the Early Healthy Lifestyles (EHL) risk assessment tool; and 3) data integration into child’s electronic health records with display and documentation features to inform counseling and coordinate care between pediatric PCPs and WIC nutritionists. The primary study outcome is rapid infant weight gain from birth to 6 months derived from sex-specific World Health Organization adjusted weight-for-age z-scores. Additional outcomes include care coordination, messaging consistency, parenting behaviors (e.g., food to soothe), self-efficacy, and infant sleep health. Infant temperament and parent depression will be explored as moderators of RP effects on infant outcomes.

**Discussion:**

This pragmatic patient-centered RP intervention integrates and coordinates care across clinical and community sectors, potentially offering a fundamental change in the delivery of pediatric care for prevention and health promotion. Findings from this trial can inform large scale dissemination of obesity prevention programs.

**Trial registration:**

Restrospective Clinical Trial Registration: NCT03482908. Registered March 29, 2018.

## Background

Obesity is a widespread and expensive public health problem that often begins early in life, with prevalence rates higher for economically disadvantaged children, [1–3] placing them at increased risk for future health disparities and later obesity [4–6]. Despite national and federal initiatives to reduce obesity among low-income children, the prevalence is higher (14.5%) in 2- to 4-year-old children enrolled in the Special Supplemental Nutrition Program for Women, Infants, and Children (WIC) than in nationally representative populations (8.9%) [7]. In fact, there has been an upward swing in severe obesity among children aged 2- to 5-years [8]. Together, these data and others have resulted in a series of reports calling for a coordination of efforts locally, between primary care and community-based programs like WIC, to enhance childhood obesity prevention [9–15]. Yet, there is a lack of promising interventions that test coordination strategies between clinical and community settings to prevent early childhood obesity [16].

Pediatric primary care providers (PCPs) and WIC nutritionists are viewed as credible and trustworthy sources of parenting and feeding information by mothers, and the timing of well-child visits and WIC appointments overlap during a child’s first year. Therefore, there are many opportunities for families to receive nutrition and obesity preventive counseling and to deliver consistent, coordinated care [17]. According to the American Academy of Pediatrics (AAP) Recommendations for Preventive Pediatric Health Care, infants should attend 5 well-child visits in the first 6 months of life [18]. Similarly, infants enrolled in the WIC program, administered by the United States Department of Agriculture, [19] are recommended to attend 3 visits with a nutritionist during the first 6 months of life to receive nutrition education, health care referrals, and breastfeeding support or supplemental formula. Despite this, data indicate that the education messages related to obesity prevention are not consistent nor coordinated between pediatric PCPs and WIC nutritionists. This can result in conflict in messaging and confusion among WIC mothers, [20–23] which are serious barriers to patient-centered care and parent adoption of endorsed behaviors to promote healthy child growth [23].

The Chronic Care Model [24] provides an alternative care delivery model that advances the concept of connectivity among clinical and community health services to improve patient-centered care. Two frameworks, the Culture of Health Action, [25] developed by the Robert Wood Johnson Foundation, and an Integrated Framework to Optimize the Prevention and Treatment of Obesity [15, 26] have embraced this concept to achieve health equity and population health goals. Both of these frameworks focus on strengthening integration of health services and systems (e.g., public health, clinical, and community) to break down siloes and to engage and empower patients and their families to optimize health outcomes. The AAP has called for the integration and coordination of care between clinical health care and community settings, such as WIC, that is centered on the comprehensive needs of the patient and family leading to a reduction in fragmented, inconsistent care [27].

Advanced health information technology (HIT) strategies offer a potential pathway for successful, patient-centered obesity prevention [28, 29]. Specifically, HIT strategies provide opportunities for data exchange, integration, and sharing. Clinical pediatric PCPs and WIC nutritionists collect and electronically document anthropometric (i.e., length, weight) and behavioral (i.e., dietary) assessments to evaluate nutritional status and growth and education provided during routine visits. Both pediatric PCPs and WIC community nutritionists conduct these assessments to evaluate nutritional status and growth, and are required to provide education during visits using electronic systems to document their assessments and care. This presents an opportunity to test the impact of data sharing and coordinating care between the clinical and community settings to promote and inform personalized, evidence-based, behaviorally-anchored educational messages for patients and their families.

Existing systems provide opportunity for data sharing and coordinated care between providers that could improve consistent messaging between clinical and community settings to prevent childhood obesity. Pediatric PCPs, WIC nutritionists, and parents of infants and toddlers supported sharing health assessment data and integrating health services as strategies to improve patient-centeredness, decrease confusion, reduce care inefficiencies, and enhance quality of care as assessed in semi-structured focus groups and interviews [23]. All stakeholder groups were concerned about security and confidentiality that informed the study team’s approach to consent and secure data transfer systems in this pragmatic randomized control trial [23].

Pragmatic efforts to integrate clinical and community settings for childhood obesity prevention are needed. We propose to integrate features of an effective, home-based intervention that has been shown to impact components of a responsive parenting (RP) framework, and use this to inform a pragmatic trial. For example, The Intervention Nurses Start Infants Growing on Healthy Trajectories (INSIGHT) study was designed to promote infant self-regulation within a RP framework that included feeding, sleeping, soothing, and interactive play; this intervention program encouraged shared parent-infant decision making [30]. INSIGHT successfully reduced rapid weight gain during the first 6 months after birth and overweight status at age 1 year [31]. INSIGHT and other interventions testing RP strategies for obesity prevention were designed to test multicomponent, nurse-delivered RP guidance in ideal circumstances with manualized, relatively inflexible curriculum. In addition, these interventions were usually delivered by experienced research nurses who were trained and monitored to achieve high compliance, often with a history of success delivering similar curricula. A limitation of these trials is that they may be difficult to disseminate to large populations[32].

The aim of WEE Baby Care is to compare standard of care (control) to a RP intervention [31] to reduce rapid infant weight gain by promoting RP and infant self-regulation. Our model for the RP intervention integrates data and coordinates clinical and community care to reduce conflict in RP messaging. RP is promoted by engaging mothers in self-assessment of parenting practices that potentially place a child at risk for rapid weight gain [15, 26]. Clinical and community providers will use shared and integrated data, including the risk assessments and documentation of education, to provide tailored, consistent patient-centered care which is expected to build a supportive social context for learning and behavior change. The RP educational messages will teach mothers to use prompt, contingent, and developmentally appropriate responses to infant needs [33, 34]. HIT strategies used to share and integrate data will be described. This is unique from previous trails because it will answer the question whether a RP intervention that is coordinated between clinical and community settings can work when delivered in usual settings, under usual conditions by clinicians and community service workers [32]. We hypothesize that mother-infant dyads randomized to the RP intervention group will report greater consistency of messages between settings, impacting maternal self-efficacy, parenting behavior(i.e., shared parent-infant decision making), and infant self-regulation to prevent rapid infant weight gain compared with participants receiving standard care. We will also explore how infant temperament and maternal depression moderate the relationship between parenting behavior and infant weight gain.

## Methods

The WEE Baby Care study is a pragmatic, randomized clinical trial (RCT) that was implemented in Luzerne County of northeastern Pennsylvania. This area is characterized by the Health Services and Resources Administration in 2013 as Medically Underserved with shortages in Health, Dental, and Mental Health Professionals and having a diversity of population densities including both urban and rural municipalities. Data derived from electronic medical records at Geisinger describing pediatric patients in Luzerne County revealed that more than 30% of the patient-population self-identified with a racial/ethnic minority group and 44% received Medical Assistance (proxy for low-socioeconomic status) in 2013, indicating the potential to reach families experiencing health disparities. This study was approved by the Institutional Review Boards of Geisinger, a large integrated health system, and The Pennsylvania State University.

### Sample size

The primary outcome for this trial is rapid weight gain derived from World Health Organization sex-specific weight-for-age z-scores from pre to post-intervention. We define rapid weight gain score as the standardized residuals from the linear regression of WAZ at 6 months on WAZ at birth, adjusting for length-for-age z-scores at birth and 6 months, and infant age at the 6 month time period. A score greater than zero would indicate a child with greater than average weight gain, which we define as rapid weight gain. A score less than 0 would indicate a child with slower weight gain from birth to 6 months. Additionally we will examine weight-for-age z-scores at the final outcome measure, to determine if the RP intervention children have a lower average WAZ score than the control children. Using similar rapid weight gain data, with effect size = 0.37, power = 0.80, and 5% Type I error, we will need 116 subjects/arm, for a total of 232 subjects in this 2-armed study (SAS, version 9.4). For this 6–7 month-long project, we estimate an 80% retention rate and will recruit 290 mother/infant dyads. In addition, the study is powered to detect a 15% reduction in the use of food to soothe among RP intervention compared to control mother-infant dyads. To detect this difference with 80% power and a 5% Type 1 error, 290 participants are required.

### Participants

**Fig. 1:**
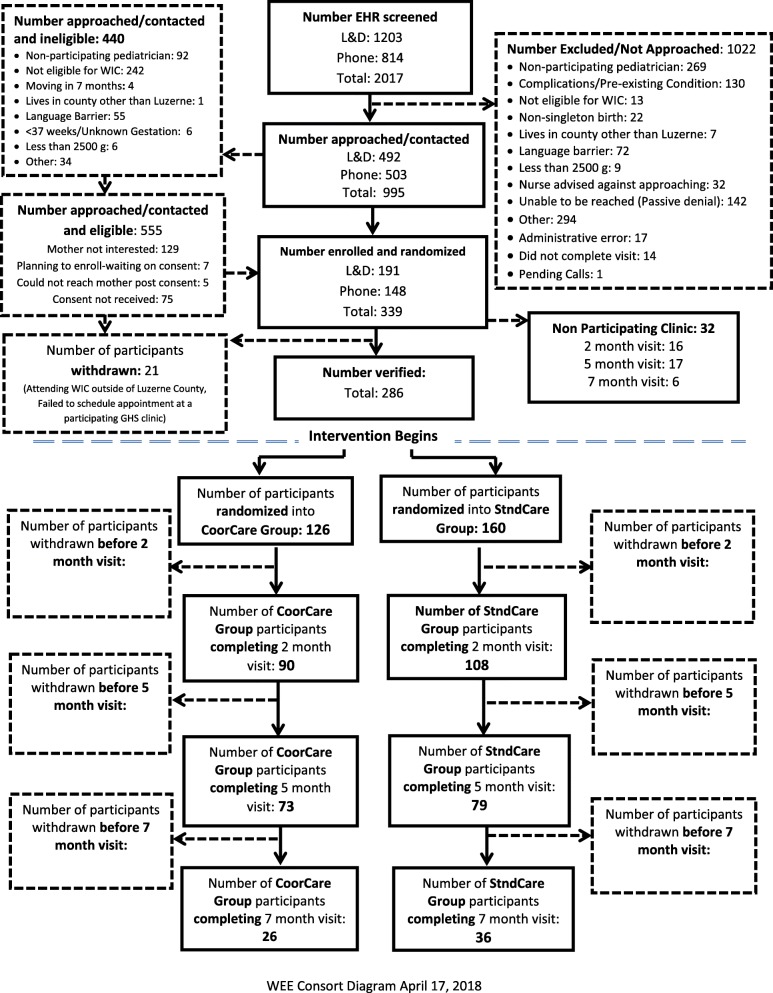
WEE Baby Care CONSORT as of 4/17/18

Eligible mother-infant dyads include full term (≥ 37 weeks gestation), singleton newborns delivered to English-speaking mothers greater than 18 and less than 55 years of age, who will be seen (or intend to be cared for) by a participating pediatric PCP in a participating Geisinger pediatric clinic (8 clinics), and who are enrolled, or are eligible to enroll, in the WIC program. Depite completing the training sessions, one clinic did not implement any component of the intervention and thus participants randomized to intervention at that clinic were dropped as noted on the consort diagram(Fig. 1). Mother-infant dyads are excluded if there is a plan for the newborn to be adopted, if the mother anticipates switching to a non-participating provider within 6–9 months, if the mother-infant dyads do not live in the service area of the participating WIC clinics, if the newborn’s birth weight is < 2500 g, or if either mother or infant has significant health issues that would affect study participation or feeding and/or growth (e.g., major depression, substance abuse, infant cleft pallet, failure to thrive).

### Recruitment

A multipronged approach will be used to recruit participants who intended to receive or received care from a participating Luzerne County pediatric clinic within Geisinger and who are also enrolled or eligible for participation at a WIC program within the defined geographic region. Of the four recruitment strategies, two strategies will use active methods and two will use passive strategies to identify potential participants. Active methods use aggregate consumption of electronic health record (EHR) data. The first strategy identifies potentially eligible mother-infant dyads in Labor and Delivery based on available eligibility criteria pulled from EHRs. These mother-infant dyads are approached in the maternity ward by a trained research staff member who explains the study and screens for eligibility. The second strategy also utilizes EHRs to identify eligible mother-infant dyads who were not approached in the Labor and Delivery unit (e.g., mother was in shower or sleeping, mother delivered at another hospital) but had a well-child visit scheduled at a participating pediatric clinic. Clinic staff distribute a study flyer to these mothers at the infant’s well-child visit. After the well-child visit was completed, research staff contact mothers by phone to explain the study and complete eligibility screening. Two additional passive strategies include posting signage at WIC clinics to recruit women prenatally and a Facebook advertisement campaign targeting women who potentially met eligibility criteria (e.g., geographic location, socio-economic status, childbearing age) created by Geisinger’s communication team.

### Study flow

After screening is complete and eligible participants complete the electronic consent form, they enrolled into the study. Mothers report demographic information at baseline and mother-infant dyads are randomized within 35 days of delivery to either the RP intervention group or the standard of care control group with stratification on birth weight for gestational age (<50th percentile or ≥ 50th percentile), infant race, and parity (primiparous or multiparous). Next, initial data sharing occurs for initial verification of each mother-infant dyad. Research staff transfer patient data to WIC through a secure file transfer protocol site. The Pennsylvania WIC Program connects to the file transfer protocol site with a secure username and password to automatically download these data and verify WIC participation. Verification for enrollment occurs if the participant has a scheduled WIC appointment. Participants have to meet federal requirements (e.g., income verification, nutrition risk, etc.) for WIC participation as determined by staff in the WIC clinics. After WIC participation is verified, the maternal-infant dyads are enrolled in the study. For the intervention group, daily data sharing is enabled WIC and Geisinger at the dyad-level and intervention materials are mailed.

### Description of WEE baby care study intervention components

#### Responsive parenting intervention group

**Fig. 2:**
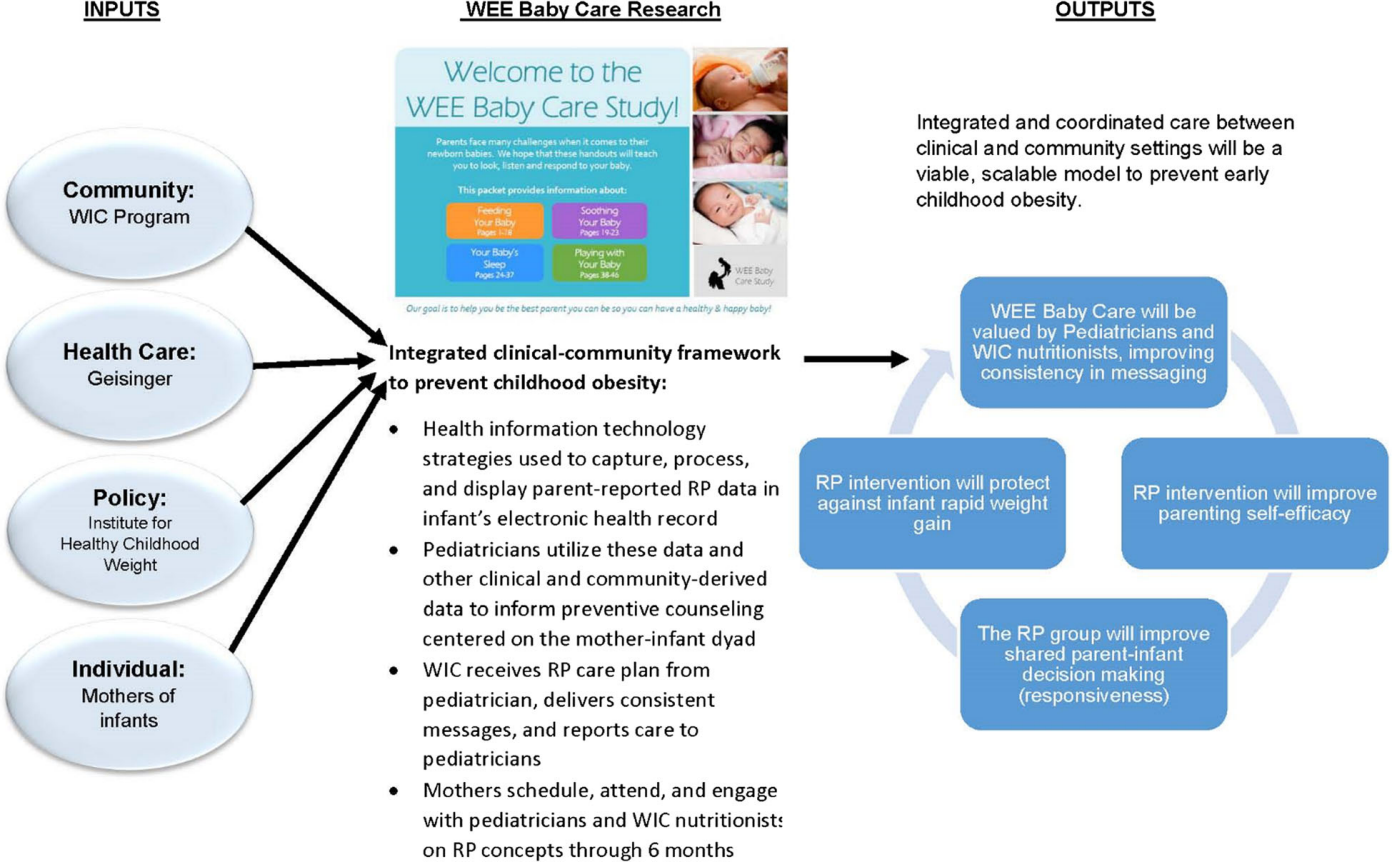
An illustration of a framework that integrates clinical and community systems to prevent and manage obesity

An illustration of the WEE Baby Care framework that integrates clinical and community systems to prevent and manage obesity is shown in Fig. 2. Inputs into WEE Baby Care included the community and health care settings, policy stakeholders, and individuals (i.e., mother-infant dyads). Mother-infant dyads in the RP intervention group are exposed to 3 intervention components: 1) RP curriculum training for pediatric PCPs and WIC nutritionists and RP materials delivered by WIC nutritionists during regularly scheduled appointments; 2) parent-reported data using the Early Healthy Lifestyles (EHL) risk assessment tool; and 3) data integration into child’s health records with display and documentation features to inform counseling and coordinate care between pediatric PCPs and WIC nutritionists. The outputs of WEE Baby Care are to integrate and coordinate care between clinical and community settings to develop a viable, sustainable model to improve messaging between providers, improve parent self-efficacy and shared parent-child decision making for the prevention of infant rapid weight gain.

##### Component 1: Responsive parenting curriculum

The WEE Baby Care RP curriculum was informed by the AAP Healthy Active Living for Families (HALF) curriculum [35] in conjunction with selected messages from the INSIGHT study [30]. Age-appropriate, anticipatory messages are written at a 5th grade reading level using simple terms and frame RP messages that would resonate with parents (i.e., how to get baby to sleep through the night, how to calm a fussy baby). Simple explanations of “why” were provided to help support the suggested behavior changes. Messages are organized into four categories: 1) Feeding your baby, 2) Soothing your baby, 3) Your baby’s sleep, and 4) Playing with your baby. Target behaviors can be seen in Table 1. Intervention participants receive these RP messages throughout the study through several delivery modes: (1) handouts delivered by mail following randomization; (2) from pediatric PCP at each well-child visit, and (3) from WIC nutritionists during regularly scheduled visits at multiple time points throughout the study. For the mailed curriculum, participants receive a welcome packet specific to treatment (i.e., RP intervention or control) that includes information about what to expect in the first few days with their newborn directly following randomization. The RP intervention group also receives a more comprehensive packet with RP curriculum that includes anticipatory guidance arranged by development from birth to 8 months that is mailed. Lastly, WIC nutritionists disseminated and discussed the same RP curriculum at the infant intake, 3 and 6 month WIC appointments.

**Table 1:** Example of WEE Baby Care RP messages delivered by WIC nutritionists and pediatricians

Feeding your baby	Soothing your baby	Your baby’s sleep	Playing with your baby
Breastmilk, formula, other beverages (water, cow’s milk, juice guidance)	Baby’s temperament	Amount of sleep needed (day and night)	Play is essential for development; fun activities and games to play with baby to support motor and social skills
Bottle feeding, including what not to put in bottle (cereal, juice)	Reasons for crying (not only hunger);	Sleep safety/SIDS prevention	Tummy time tips
Hunger and fullness cues	Expectation for amount of daily crying	Bedtime before 8:30 pm	Limiting time in restrictive baby gear (car seats, carriers, strollers, swings, etc.)
When and how to introduce solids, including what not to serve	Methods for soothing baby: swaddling, holding on side or belly, rocking or swaying, shushing, giving a pacifier	Bedtime and naptime routines (don’t make feeding last step)	Spend time outdoors
Shared responsibility of feeding		Putting baby down drowsy but awake; avoid feeding or rocking to sleep	Limiting screen time
Repeated exposure to foods		No television at bedtime/no TV where baby is sleeping	Modeling – reducing own screen time and connecting with baby
Avoid controlling feeding practices (pressure, restriction)		What to do when baby wakes at night; responding differently day vs. night	
Self-feeding (cup, finger foods)		Sleep disruptions during developmental milestones	

##### Component 2: Early healthy lifestyles (EHL) risk assessment tool

The EHL risk assessment tool is a behaviorally-based instrument that allows mothers to self-assess infant feeding and other parenting practices that may be associated with their children’s risk for obesity and obesogenic behaviors in the future. Questions on the tool center on infant’s food and beverage intake in the past week, parental soothing practices, sleep hygiene, the infant activity, media behavior, and environment. The tool was developed using the Centers for Disease Control and Prevention’s Infant Feeding Practices Study II [36, 37], the INSIGHT trial, [30] and HALF materials [35]. The EHL risk assessment tool is triggered by each scheduled well-child visit to capture time-sensitive developmental changes. The tool is delivered through the Geisinger patient portal for collection of parent-reported data and implemented as standard of care in most pediatric clinics. In the event that a parent did not complete the EHL prior to arriving at the visit, clinic staff are oriented to encourage parents to complete in the waiting room prior to being roomed for the well-child visit. In the present study, EHL risk assessment tool will be suppressed for children in the control condition. The EHL risk assessment tool can be completed in approximately 2 min and these patient-reported data are immediately integrated (and stored) into the child’s EHR. These data identify relevant gaps in parent knowledge and behaviors that are mapped to the RP curriculum. Pediatric PCPs and WIC nutritionists receive training from study staff on the RP curriculum and EHL. Pediatric PCPs and WIC nutritionists provided input on the display of EHL data as decision support in their respective electronic documentation systems. For example, pediatric PCP use is optimized by using the Epic® EHR functions that display patient-reported EHL data alongside RP talking points and features to facilitate documentation in the progress note. Standard and novel WIC nutrition education codes were created and are mapped to the EHL talking points to facilitate documentation within the participant management system. The intent of this component is to enable pediatric PCPs and WIC nutritionists to deliver patient-centered care responsive to dyad-level risk behaviors, reinforce the RP curriculum, and document education messages to share with the other setting.

##### Component 3: Data integration for the coordination of care between clinical and community settings

**Fig. 3:**
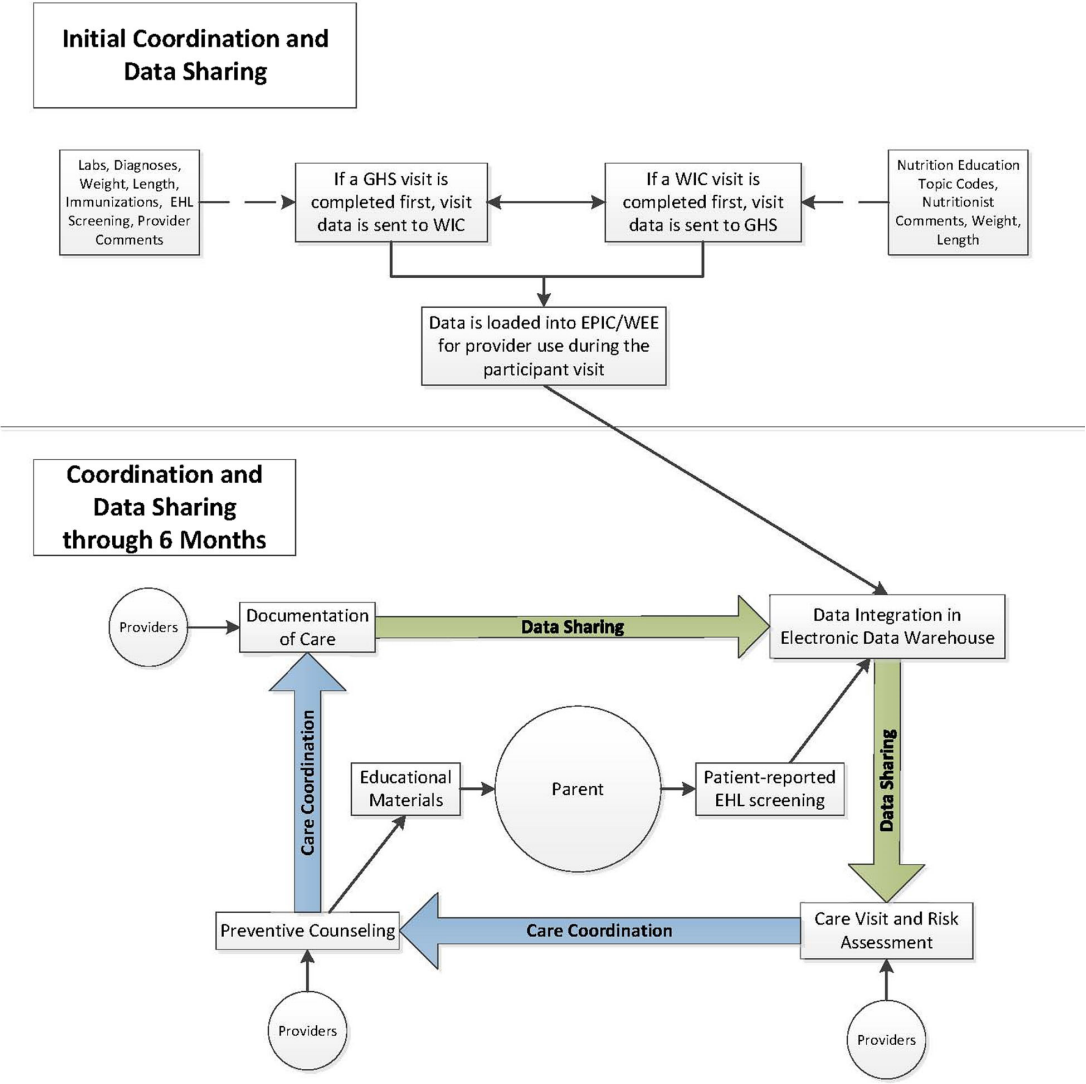
WEE Baby Care flow chart for care coordination and data sharing

Following the well-child visit, the patient-reported data from EHL risk assessment tool and clinical encounter data are shared and integrated at the dyad-level with WIC nutritionists using their parallel electronic participant management system to inform tailored RP education (see Fig. 3). Subsequently, WIC nutritionists interact with the EHL data and clinical care records, document the education they provided locally, and communicate with the pediatric PCP to inform the next well-child visit. All data sharing is automated, bi-directional, and continuous to respond to the real-world patterns of care across sectors.

Care is coordinated between 3 parties in the WEE Baby Care study – parent and two types of providers (pediatric PCP, and WIC nutritionist) - to create a communicative and supportive team. As shown in Fig. 3, an opportunity for care coordination exists when there is an electronic exchange that transfers data between these parties (e.g., parent completes EHL and data is transferred to health system’s data warehouse; pediatric PCP uses data to inform and document care and transfers data to WIC; and then WIC nutritionist uses data to inform and document education and transfers data back to the pediatric PCP to restart the cycle with current, time relevant EHL data). The term “transfer” is used to describe a HIT strategy of automating secure, electronic sharing of agreed-upon data elements but the term “communicate” could be used interchangeably with transfer to represent messages being shared from one party to another (see Table 2). Care is coordinated when the receiving party interacts with the data (or message) from the other setting and then documents the care the receiving party provided locally. By coordinating care, pediatric PCPs and WIC nutritionists have the ability to tailor preventive messages to the needs of the mother-infant dyad based on EHL data, and communicate care plans with one another to reinforce and enhance consistent RP messages.

**Table 2:** Data elements shared by Pediatric PCPs with WIC nutritionists and vice versa

Geisinger Data Elements	WIC Data Elements
Demographics Anthropometrics Hemoglobin Hematocrit Breastfeeding Status Formula use Pediatric PCP education topic codes Pediatric PCP comments to WIC nutritionists Encounter/problem list diagnoses Immunizations Early Healthy Lifestyle risk assessment data	Demographics Anthropometrics Hemoglobin Hematocrit Breastfeeding Status Formula use Nutrition education topic codes WIC nutritionist comments to Pediatric PCP

Given that a series of interactions is expected through the natural progression of well-child visits and WIC appointments, supplemented by the EHL assessments, there are multiple opportunities for care coordination of RP messages that can dynamically evolve with the mother-infant relationship during a rapid developmental period, i.e., the first 6 months of the infant’s life. For example, the EHL data collected at each well-child visit can be used at the following WIC visit by the nutritionist to tailor the RP messages to meet needs of the mother-infant dyad and discuss specific messages from the RP curriculum packets. WIC nutritionists discuss the needs of each mother-infant dyad using the EHL data to discuss specific RP messages from a RP curriculum packet relevant to birth to 3 month infants (to discuss at the infant intake appointment) and an additional packet relevant to 3 to 8 month old infants (to be discussed at approximately the 3 and 6 months WIC appointments).

The process of data sharing precedes care coordination and is possible given that Geisinger and the Pennsylvania Department of Health and Human Services (i.e., WIC) each maintain electronic patient and client management systems, respectively, with informed consent from mothers and an executed data sharing agreement between institutions. Functionally, a secure file transport portal is utilized for bi-directional data sharing on enrolled study participants. For example, upon enrollment into the study at Geisinger, a mother and her child’s demographic information (Study Identifier, Name, Date of Birth, Telephone Number) are uploaded to the Geisinger secure file transport portal site. Next, the WIC state agency office that maintains all client data automatically sends a notification email to local WIC staff that triggers staff to contact the participant to schedule a WIC visit (newborn intake appointment). Once a WIC visit is scheduled, the local WIC staff verified WIC participation and data were automatically pushed back to Geisinger via the secure file transport portal. Geisinger staff then enabled data sharing for care coordination for participants randomized to the intervention arm and suppressed EHL data collection in pediatric care for those randomized to the control arm. Data are shared daily using the secure file transport portal and electronic patient and client management systems at Geisinger and WIC, respectively, are refreshed with these data within 24 h to inform patient-centered and coordinated care. For example, after a completed WIC visit, data from the participant’s visit regarding RP education is shared via the secure file transport portal with Geisinger. At Geisinger, the WIC visit information is integrated into the child’s EHRs a data table to directly align with the patient-reported EHL data and RP talking points. The pediatric PCP can interact with these data and “check-off” talking points that are discussed with the parent and automatically generate progress note documentation.

### Standard of care control group

Mother-infant dyads randomized to the control group will receive standard of care. Pediatric PCPs and WIC nutritionists will not have access to participant EHL data in the EHR, mothers will not receive the WEE Baby Care handouts, and there will be no coordination of care between well-child and WIC visit settings using HIT strategies. Once WIC verified participation in the WIC program, participant IDs of those randomized to the control group are hidden in the IT infrastructure to prevent contamination.

### Measures

Anthropometric data and self-report survey data are collected on all participants when infant is less than 3 weeks old, and again at approximately 2, 4 and 6 months of age. Mothers self-report on self-efficacy, home environment, feeding and parenting practices, food insecurity, infant and maternal sleep, maternal depression, infant temperament, and perceptions of coordinated care and patient involvement in care at Geisinger and WIC. As shown in Table 3, many measures are repeated across three or four time points. Mothers are asked their preferred method for completing surveys, either online through an emailed link to an electronic survey system [38] or a hard copy sent by mail with a prepaid return envelope, and survey completion was incentivized with gift cards.

**Table 3:** WEE Baby Care study measures

Construct	Time Point (Infant Age)			
	Time of enrollment (birth to 21 Days)	Between 2 and 3 Months	Between 4 and 5 Months	Between 6 and 7 Months
Infant anthropometrics (retrieved from electronic records at WIC and Geisinger)	X	X	X	X
Obesity risk (Early Healthy Lifestyles Tool)	X	X	X	X
Screening and eligibility	X			
Parenting self-efficacy		X	X	X
Feeding attitude and beliefs		X	X	X
Food to soothe distress		X	X	X
Responsive parenting style			X	
Food insecurity				X
Infant sleep health		X	X	X
Maternal sleep		X	X	X
Maternal depression			X	X
Infant temperament			X	
Maternal perceptions of coordinated care			X	X
Maternal perceptions of involvement in care				X
Coordination of care: shared documentation of visit content and care plan (objective assessment; log of bidirectional data flow)		X	X	X

## Discussion

WEE Baby Care fosters parent education and decision making about early infant feeding through coordinated clinical and community care linked by shared EHR education and messaging. Continuous engagement of mothers in risk assessment and the integration of these data to tailor and coordinate care across clinical and community sectors is innovative. This is done using advanced HIT strategies to deliver a patient-centered RP intervention. In this trial, trusted providers (pediatric PCPs and WIC nutritionists) are trained to deliver an evidence-based curriculum for the prevention of infant rapid weight gain via a multi-component intervention in settings where counseling and patient education is typically delivered, demonstrating sustainability. Thus, WEE Baby Care will test the feasibility of delivering a patient-centered intervention that is integrated and coordinated, and its effectiveness under the usual conditions in the settings that the program is delivered in (i.e., pediatric clinic and WIC office). If effective, this program could serve as a model for dissemination. Another strength of WEE Baby Care is that it included a transdisciplinary team of experts, including researchers and clinicians who developed the intervention, and most important, key stakeholders including the AAP and the Pennsylvania WIC Director of Nutritional Services to inform future scalability. Unique from other trails, WEE Baby Care opened communication utilizing data sharing and documentation between clinical and community sectors thereby creating a supportive environment for behavioral change to enhance the opportunity to achieve population health goals that aim to prevent and reduce pediatric obesity.

## Conclusion

Early infant accelerated weight gain has been linked to later obesity and efforts to leverage and coordinate care in the first 6 months of life by integrating clinical and community systems are likely to offer the best opportunities for patient-centered childhood obesity prevention to advance population health objectives. It is well-established that pediatric PCPs and WIC nutritionists share the common goals to improve nutrition and increase physical activity to prevent obesity despite barriers such as limited time, training/skill, resources, and lack of parent interest.. Utilizing the Integrated Framework to Optimize the Prevention and Treatment of Obesity, [15, 26] advanced HIT strategies provide a solution to these challenges and barriers to improve quality of care. Developing systems for integrated, coordinated care and communications between clinical and community settings could be an effective and efficient way to decrease conflict in messaging, increase dose, and reinforce education to optimize early-life intervention on parenting behavior and child health outcomes.

## Abbreviations

EHL: Early Healthy Lifestyles
HALF: Healthy Active Living for Families
HIT: health information technology
PCP: Primary Care Provider
RP: Responsive parenting
WIC: The Special Supplemental Nutrition Program for Women, Infants, and Children Program
